# Tailoring the emission color in Sm^3+^/Li^+^-doped calcium hydroxyapatite nanocrystals: a path toward cryogenic luminescent thermosensors

**DOI:** 10.1038/s41598-026-45561-7

**Published:** 2026-03-29

**Authors:** Paulina Sobierajska, Rafal J. Wiglusz

**Affiliations:** Institute of Low Temperature and Structure Research, Polish Academy of Sciences, Okolna 2, 50-422 Wrocław, Poland

**Keywords:** Calcium nanohydroxyapatite, Lithium(I) and samarium(III) ions, Nanosized thermometer, Cryogenic applications, Charge compensation, Chemistry, Materials science, Nanoscience and technology

## Abstract

This study reports the synthesis and physicochemical characterization of Sm³⁺, Li⁺ ions-co-doped nanocrystalline calcium hydroxyapatite (nHAp) as a cryo-compatible luminescent nanothermometer. This material exhibits a temperature-dependent emission color shift from blue-green at 77 K to orange-red at 300 K, with a high relative sensitivity (a maximum of 0.025 K^−^¹ at 200–225 K). The presence of a broadband self-activated emission band originates from the host lattice and is related to charge-compensation phenomena. Due to the linear color tuning below room temperature, the obtained materials enable precise, real-time temperature monitoring under cryogenic conditions. This type of nanosized thermometer could be particularly useful in cryopreservation, enabling accurate control of freezing and thereby improving the survival of stored biological materials.

**Graphical Abstract:**

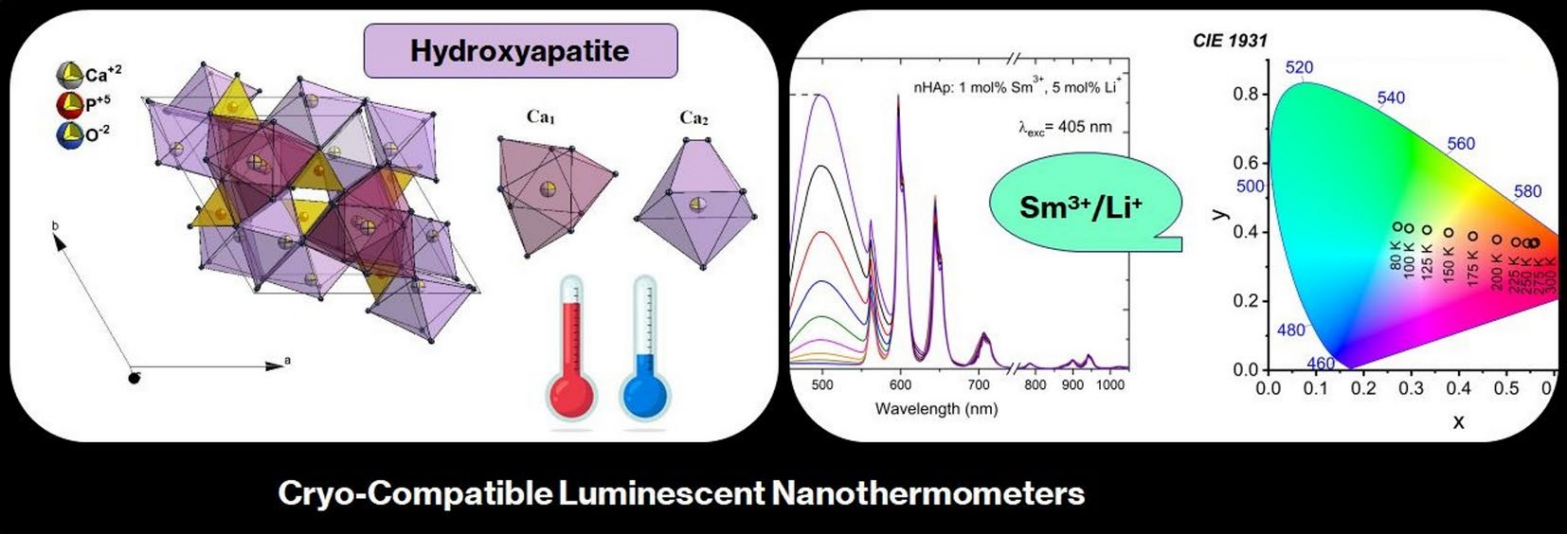

## Introduction

Phosphor materials activated by rare earth ions have potential in various technologies, including lighting, displays, and biomedical applications, due to their unique luminescent properties. Rare-earth cations are characterized by narrow, intense emission bands and long emission lifetimes^1,2^. Their unique optical properties arise from shielding of the 4f electrons in the RE^3+^ ions by the outer 5s^2^ and 5p^6^ shells. Consequently, the *4f–4f* transitions are only weakly dependent on the surrounding crystal field, resulting in relatively stable emission characteristics. Nevertheless, the crystal lattice remains a significant determinant of luminescence efficiency. Therefore, researchers have explored various strategies to improve optical properties, including modifying the crystal structure through cationic and anionic substitutions, optimizing energy transfer, and modifying crystallographic sites. These approaches modulate the local environment of the active ions, thereby affecting symmetry, phonon energy, and nonradiative relaxation pathways, ultimately improving luminescence properties.

A representative example is the Sm^3+^ ion, which has a *4f*^*5*^ electronic configuration and is designated as a Kramer ion, because its electron states are doubly degenerate for any crystal field perturbation^3^. The maximum number of Stark components for a Kramer ion with a ^*2 S+1*^*L*_*J*_ state is *J + ½* manifold for any symmetry lower than cubic^4^. Moreover, the compounds associated with Sm^3+^ ions exhibit narrow-line emission profiles and long lifetimes and can be used as labels in multi-analyte assays^5^. Furthermore, of great interest is the use of these compounds at the nanometer size, when phosphor nanoparticles have significant Stokes shifts (i.e., significant energy separation between excitation and emission), narrow line-shaped emission bands, long-lasting luminescence (about 1–2 ms), and inherent photostability^6^.

Apatite-based materials are highly desired as host materials because they are easy to synthesize,  cost-effective and biocompatible. Hydroxyapatite (Ca_10_(PO_4_)_6_(OH)_2_ labelled as HAp) has two types of Ca^2+^ sites: nine-coordinated *4f* site with *C*_*3*_ point symmetry and seven-coordinated *6 h* site with *C*_*s*_ point symmetry. It has a strong affinity for Ca^2+^ ion-substitution by other cations, mainly Ag^+^, Cu^2+^, Zn^2+^, Li^+^, Na^+^, K^+,^ or RE^3+^ ions, which in turn are widely used to develop new compounds with specific properties^7^. The ability of HAp to incorporate ions of different oxidation states requires charge compensation to maintain electro-neutrality, and this must occur without significant alterations in its structure. This compensation process involves the formation of vacancies or coupling ions of different charges. Our previous research describes the charge-compensation mechanism for HAp nanoparticles doped with rare-earth ions in which Ca^2+^ ions are co-substituted by Eu^3+^ and Li^+^ ions^8^. Based on the Judd-Ofelt theory, we found that co-doping apatite with Li^+^ ions significantly affects the quantum efficiency of Eu^3+^ in this system, reaching up to 28%. The addition of Li⁺ ions to 2 _mol_% enhanced the luminescence of Eu³⁺ ions by inducing local structural distortions around the Ca_1_ site, increasing the asymmetry of the Eu³⁺ ion coordination environment, and promoting stronger electric-dipole transitions. As a result, the luminescence efficiency increased from 62% in the Li-free material to up to 90% with 2 _mol_% Li^+^ added. At higher Li⁺ ion concentrations, Eu³⁺ ion preferentially occupied the more symmetric Ca_2_ site, leading to a reduction in asymmetry and thus a decrease in the luminescence efficiency to 80%.

The intensity of emission can also be improved effectively by adding lithium ions in other phosphate matrices, such as Sm^3+^:Ca_3_Sr_3_(PO_4_)_4_:^9^. Therefore, in the present work, we focused on Sm^3+^ ions that absorb the electromagnetic radiation in the UV-visible region and emit orange-red light at about 600 nm corresponding to the ^*4*^*G*_*5/2*_ *→* ^*6*^*H*_*7/*2_ transition^2,7–10^. The Sm³⁺ ion offers several advantages over the Eu³⁺ ion used in commercial red phosphors, such as lower cost and enhanced color saturation.

The crystal lattice of HAp is stabilized by not only ionic substitutions but also by the creation of vacancies, defects, and distortions^10,11^. Such a modified host lattice can offer efficient, stable, and broadband self-activated emission in the visible spectrum. In recent years^12–14^ the blue luminescence from HAp nanoparticles, without the presence of activators, has been extensively studied. In the present work, the blue-green emission from the HAp matrix was observed and enhanced by Li^+^ ions substitution. Simultaneously, the colour of the observed emission can be tuned to the orange-red by using Sm^3+^ ions. Due to the existence of many defects in HAp structure, the primary absorption energy states responsible for radiative emissions are unclear^15,16^. Therefore, our goal was to experimentally investigate the possible origins of this self-activated broadband emission that appeared under NUV excitation below room temperature.

Another goal of this study was to determine whether the proposed material could be used as a luminescent nanothermometer^17–19^. For many years, luminescent nanothermometers have been considered due to their ability to provide non-invasive, high-resolution temperature measurements, especially in living cells^20^. Such nanothermometers work based on temperature-dependent changes in optical properties, such as emission intensity, wavelength shifts, or lifetime changes. It makes them ideal candidates for applications where it is not possible to use traditional contact thermometry. Finding how sensitive a material is to temperature change is therefore crucial for assessing its potential in biomedical applications, e.g., mapping intracellular temperature during cryopreservation or heat therapy^20–22^. Moreover, the biocompatibility of the proposed material is crucial for the practical application of these nanothermometers in cryopreservation, as it ensures that the materials do not induce cytotoxicity, immunological reactions, or chemical changes in biological samples. Biocompatible luminescent nanoparticles can be placed in close proximity to cells or tissues, enabling accurate, real-time temperature monitoring directly at the target site. This minimizes cellular stress, improving the survival and viability of preserved biological materials, such as cell lines, tissues, or organs for transplantation.

## Materials and methods

The experimental protocols were based on the methods described in our recent publications^8,10,23^, with minor modifications detailed below in this section.

### Synthesis of Ca_10_(PO_4_)_6_(OH)_2_ un-doped, as well as doped and co-doped with Li^+^ and Sm^3+^ ions

The nano-hydroxyapatite (nHAp), as well as nHAp doped Li^+^ and Sm^3+^ ions were obtained with the co-precipitation method followed by calcination at 500 °C. The doping concentrations of Li^+^ and Sm^3+^ were 1–5 _mol_% and 1 _mol_%, respectively, in replacement of the total amount of Ca^2+^ ions. In a typical synthesis of 1 _mol_% Sm^3+^ and 1 mol% Li^+^ ions co-doped HAp (Sm^3+^/Li^+^:nHAp), 0.01723 g of Sm_2_O_3_ (99,99% Alfa Aesar, USA) was dissolved in diluted HNO_3_ (65% ultranal, Avantor Performance Materials) with stirring and then was re-crystallized three times in order to obtain Sm(NO_3_)_3_. Then, 2.2862 g of Ca(NO_3_)_2_·4H_2_O (≥ 99% Acros organics), 0.0068 g of LiNO_3_ (≥ 99% Acros organics) were dissolved in deionized water and mixed with the Sm(NO_3_)_3_ aqueous solution to form a solution. Simultaneously, 0.7828 g of (NH_4_)_2_HPO_4_ (≥ 98% Avantor Performance Materials) was dissolved in deionized water. Then, both solutions were mixed. Subsequently, the pH of the obtained suspension was adjusted to 10 by using NH_3_·H_2_O (99% Avantor Performance Materials Poland S.A., Poland). A white precipitate formed. The reaction was carried out at 90 °C for 2 h. Finally, the material was isolated from the solvent as a white solid, dried for 24 h at 90 °C, and further calcined at 500 °C for 3 h to remove the amorphous phase and co-products of the synthesis.

### Material characterization

#### Structural and morphological analysis

Powder X-ray diffraction (XRPD) patterns were obtained in a *2θ* range of 5–100° with X’Pert PRO X-ray diffractometer (Cu, Kα1: 1.54060 Å) (PANalytical).

The crystallite sizes (*D*) of the studied materials were estimated from the full-width at half-maximum (*β*) of the diffraction peaks, using Debye–Scherrer’s equation:

1 $$\:D=\frac{k\lambda\:}{\beta\:cos\theta\:}$$

where *λ* is the wavelength of the X-ray radiation (0.154 nm), *k* is a constant, and *θ* is the Bragg’s diffraction angle.

Fourier transform infrared spectra (FT-IR) were recorded using a Biorad 575 C spectrophotometer in the range of 4000 to 500 cm^− 1^ in KBr pellets at room temperature. The microstructure and elemental analysis (SEM-EDS) were performed using a FEI Nova NanoSEM 230 scanning electron microscope with an energy-dispersive X-ray spectrometer (EDAX PegasusXM4). Up to 10 measurements were made. Overall chemical composition was performed by Inductively Coupled Plasma Optical-Emission Spectrometry (ICP-OES) using a Thermo Scientific iCAP 7000 spectrometer (Waltham, Massachusetts, USA).

#### Spectroscopic measurements

The UV-Vis spectra were recorded at 300 K in the back scattering mode using Agilent Cary 5000. Following the absorption reflectance measurements, the energy band gaps $\:{(E}_{g})\:$of the obtained samples were calculated by using the Kubelka–Munk function^2^:

2 $$\:F\left({R}_{\infty\:}\right)=\frac{{\left(1-{R}_{\infty\:}\right)}^{2}\:}{2{R}_{\infty\:}}=\:\frac{K}{S}$$

where *F(R*_*∞*_*)* is the reflectance function, *K* is the molar absorption coefficient, and *S* is the scattering coefficient. The relation between the *F(R)* and photon energy (*hν*) for direct and indirect optical transitions is given by:

3 $$\:F\left({R}_{\infty\:}\right)h\nu\:=C(h\nu\:-{E}_{g}{)}^{n}$$

where *n = ½* for allowed direct transitions and *n* = 2 for allowed indirect transitions, *C* is constant. It was noticed that the HAp is an indirect gap material because of *n = ½* related to the calculation of the band structure (A. Slepko and A. Demkov^2^. The optical band gap energy has been obtained by extrapolating the linear portion of the UV-Vis curves:

4 $$\:(F\left({R}_{\infty\:}\right)h\nu\:{)}^{2}=f(h\nu\:)\:$$

The excitation-emission spectra were measured using an FLS980 Fluorescence Spectrometer from Edinburgh Instruments equipped with a 450 W Xenon lamp as described in our previous paper^23^. The excitation of a 300 mm focal length monochromator was in a Czerny-Turner configuration. The excitation spectra were obtained using a ruled grating (1800 lines/mm) blazed at 250 nm. The Hamamatsu R928P side-window photomultiplier tube was used as the detector. All the excitation spectra were measured at room temperature and corrected for the detector sensitivity and excitation source intensity, respectively.

Emission spectra at room temperature as well as at 77 K were measured with a Hamamatsu PMA12 photonic multichannel analyzer. Excitation was provided by a pulsed (10 Hz) 405 nm line generated from a Ti: sapphire tunable laser pumped by the second harmonic (532 nm) of a Nd^3+^:YAG laser.

The emission spectra and decay curves as a function of temperature in the range of 77 K to 300 K were recorded with the laser diode and *λ*_*exc*_ = 405 nm. To control the temperature, samples were placed into a Linkam THMS 600 Heating and Freezing Stage. The chosen detector was a Hamamatsu PMA-12 photonic multichannel analyzer.

Decay curves at 300 K and at 77 K were collected using the tunable Ti: Sapphire laser (λ_exc_ = 405 nm) pumped by the second harmonic of the Nd^3+^:YAG: pulse laser (ƒ = 10 Hz, t < 10 ns ). To measure spectra and decay curves of the broad-band emission (λ_mon_ = 495 nm) Libra Laser Coherent (1 mJ and pulse duration equals 89 fs) was used. The excitation line 405 nm was achieved by Optical Parametric Amplifiers (Coherent). The emission spectra were recorded using a system equipped with a Princeton Instrument Acton SP2500 grating spectrograph and a Hamamatsu C5680 streak camera.

## Results and discussion

### Structural and morphological characterization of the obtained materials

**Fig. 1:**
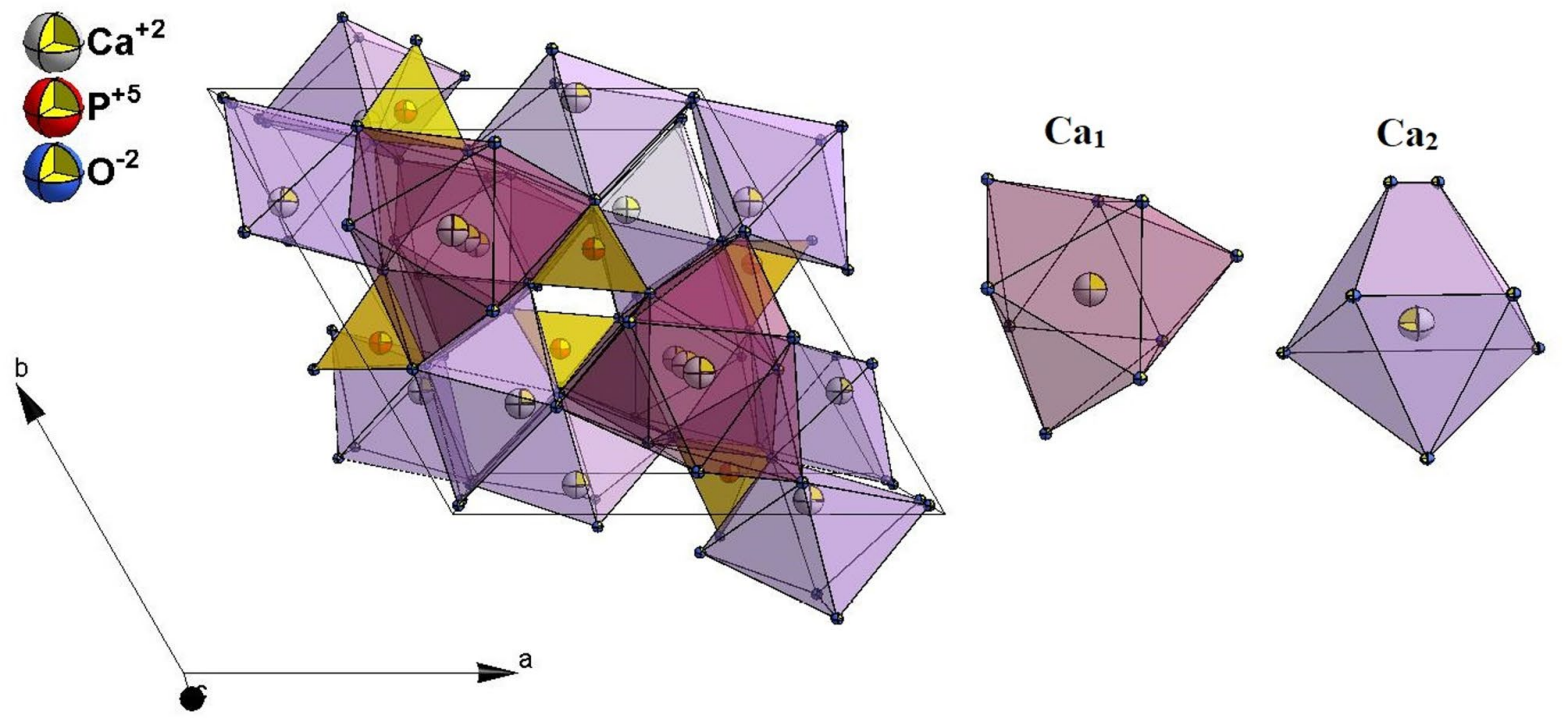
The projection of the Ca_10_(PO_4_)_6_(OH)_2_ unit cell with the Ca_1_ and Ca_2_ crystallographic positions.

The hexagonal hydroxyapatite (HAp) lattice structure and the coordination polyhedra of Ca^2+^ are presented in Fig. 1. The four Ca^2+^ ions are surrounded by nine oxygen atoms from the phosphate group, with the *C*_*3*_ symmetry site (*4f* Wyckoff position, Ca_1_ site). The six Ca^2+^ ions with a *C*_*s*_ symmetry are coordinated by seven oxygen atoms, six derived from phosphate groups and one from a hydroxyl group (*6 h* Wyckoff position Ca_2_ site). Both Ca^2+^ nonequivalent sites can be substituted by other ions with various charges as well as with different ionic radii. It is possible due to the charge compensation mechanism^11^. As an example, the substitution of the divalent calcium ions by trivalent samarium ions (Sm^3+^) leads to the charge imbalance, and two positive defects of *Sm·*_*Ca*_ are created^3^. These defects can be balanced by creating calcium vacancies (*V”*_*Ca*_) or by creating double-negative-charge interstitial oxygen (*O”*_*i*_*)*. Moreover, in the case of the *Ca*_*2*_ site, the migration of atmospheric oxygen into the lattice leads to the substitution of OH^−^ by O^2−^, and thus, the oxygen negative *O’*_*OH*_ is generated. The vacancy defects created by RE^3+^ ions could also be reduced by adding monovalent Li^+^ ions (*Li’*_*Ca*_). All these possible mechanisms of charge compensation were considered in our previous work^23^ based on Kröger–Vink notation.

**Fig. 2:**
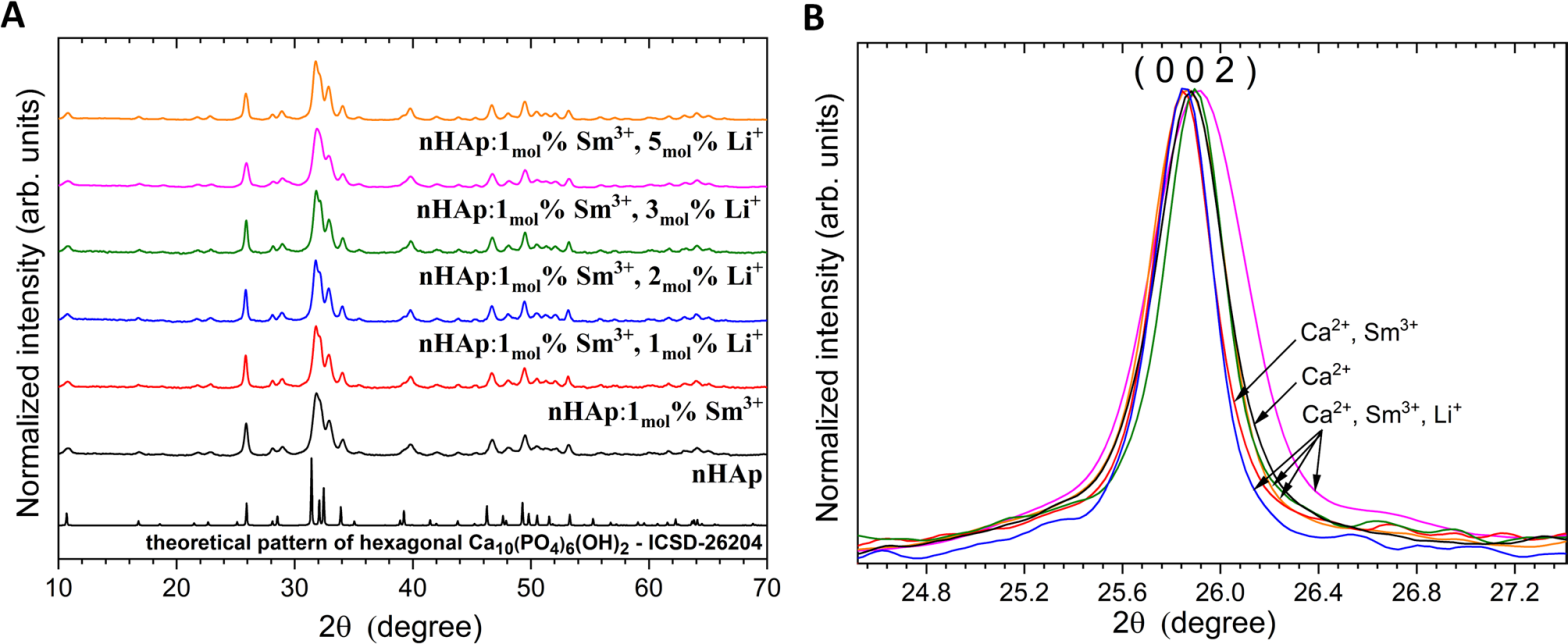
XRPD patterns (**A**) of nHAp, 1 _mol_% Sm^3+^:nHAp, as well as nHAp co-doped with 1 _mol_% Sm^3+^ and 1 _mol_% − 5 mol% Li^+^ ions, annealed at 500 °C with the highlighted (0 0 2) plane shift (**B**) induced by various types of cations located at both crystallographic positions (bottom).

The structure of the nHAp, as well as nHAp doped with Sm^3+^ ions and co-doped with Li^+^ and Sm^3+^ ions, was identified by the XRPD method. As can be seen in Fig. 2, for all samples, the diffraction peaks correspond to the hexagonal Ca_10_(PO_4_)_6_(OH)_2_ with *P6*_*3*_*/m* space group (ICSD- 26204). No other phases were detected, which suggested that the Sm^3+^ and Li^+^ ions have been fully incorporated into the host lattice of nHAp. The broadening of XRD peaks may indicate small crystallite sizes, but can also result from lattice deformations, internal stresses, or structural defects. The average crystallite size for each studied material was calculated based on Eq. 1, and the results were gathered in Table 1. Moreover, slight variations of the lattice constants and crystallite size (see Fig. 2) of nHAp were observed, especially when the lithium concentration increases.

The diffraction peak of the (0 0 2) plane is presented in Fig. 2, B. Upon doping with 1 _mol_% Sm³⁺ ions, the (0 0 2) peak shifts to a lower *2θ* value. It suggests the expansion of the unit cell. Additionally, co-doping with Li⁺ leads to a shift of the (0 0 2) peak toward higher *2θ* angles when the Li⁺ content increases from 1 _mol_% to 3 _mol_%. This observation indicates a progressive contraction of the unit cell that can be related to the incorporation of smaller Li⁺ ions (ionic radius ~ 0.76 Å). Lithium ions may occupy Ca²⁺ positions (1.18 Å at CN_9_ and 1.06 Å at CN_7_) or interstitial positions. All these results show that Sm³⁺ ions (ionic radius: (CN_9_) = 1.13 Å, Sm^3+^(CN_7_) = 1.02 Å) cause an expansion of the lattice, whereas the Li^+^ ion contracts the cell, which is also induced by increasing concentration of Li⁺ ions.

**Table 1:** Average crystallites size (D) of the Ca_10_(PO_4_)_6_(OH)_2_ (nHAp) doped and co-doped with Li^+^ (nHAp: Li^+^) and Sm^3+^ (nHAp: Li^+^/Sm^3+^), and annealed at 500 °C.

Sample	D (nm)
nHAp	36.3
nHAp: 1 _mol_% Sm^3+^	51.8
nHAp: 1 _mol_% Sm^3+^,1 _mol_% Li^+^	52.6
nHAp: 1 _mol_% Sm^3+^,2 _mol_% Li^+^	39.7
nHAp: 1 _mol_% Sm^3+^,3 _mol_% Li^+^	41.9
nHAp: 1 _mol_% Sm^3+^,5 _mol_% Li^+^	60.7

**Fig. 3:**
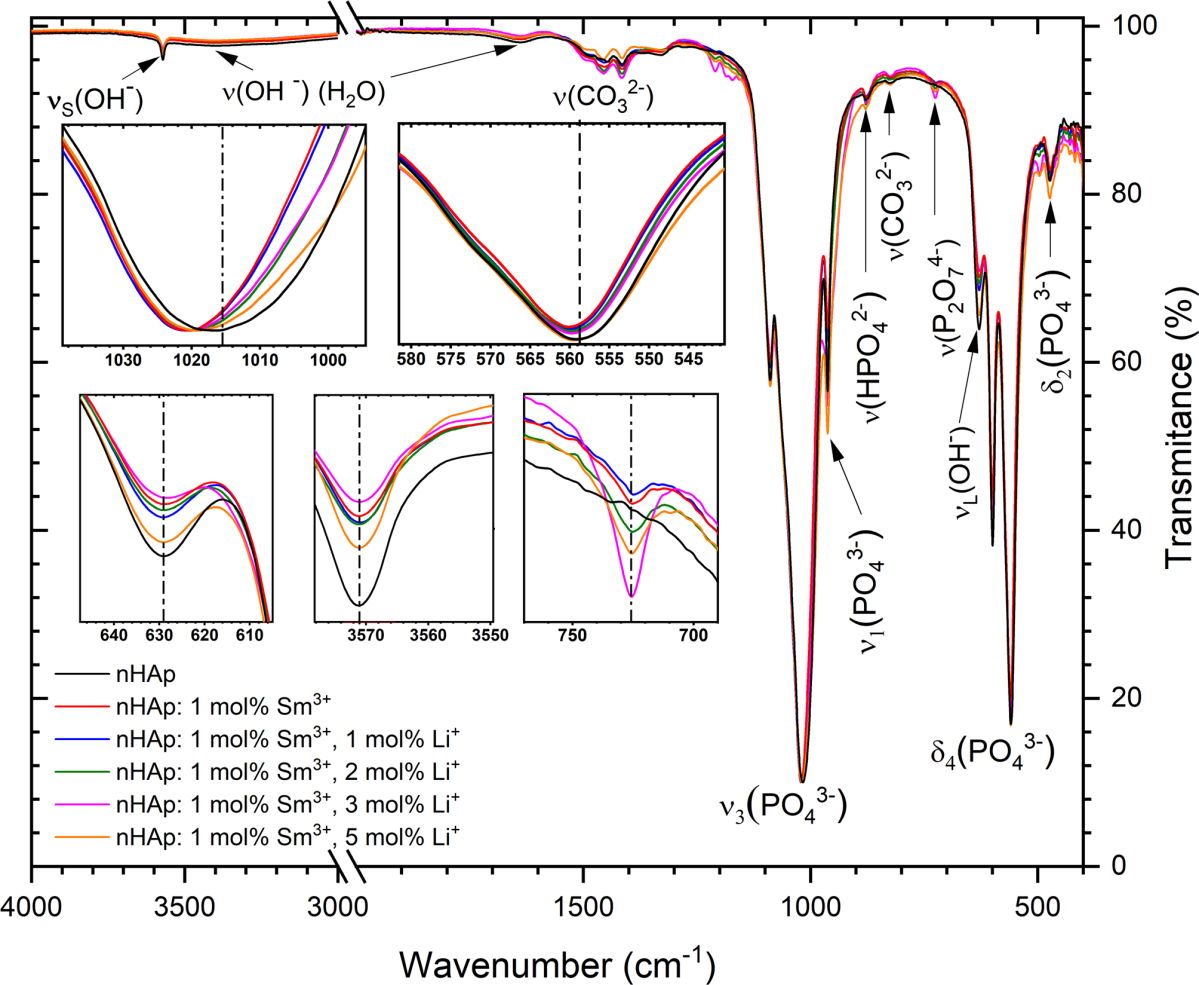
FT-IR spectra of nHAp, 5 _mol_% Li^+^ :nHAp, 5 _mol_% Li^+^, 1 _mol_% Eu^3+^ :nHAp and 5 _mol_% Li^+^ ,1 _mol_% Sm^3+^ :nHAp annealed at 500 °C.

The FT-IR spectra of the nHAp doped with Li^+^ ions, as well as co-doped with Li^+^ and Sm^3+^cations, and undoped nHAp have been shown in Fig. 3. The spectra indicate that the absorption bands at 633 and 3571 cm^− 1^ correspond to the libration motion (*ν*_*L*_) of the hydroxyl groups, reflecting restricted rotational oscillations around the O–H bond and hydroxyl stretch (*ν*_*S*_) modes of the OH^−^ ions, respectively. Whereas, the broadband at 3449 cm^− 1^ is typical of the O–H vibration of absorbed H_2_O. The 471 cm^− 1^ and 474 cm^− 1^ as well as 962 cm^− 1^ absorption bands belong to the doubly degenerate *δ*_*2*_ bending of the O–P–O bonds and non-degenerate *v*_*1*_ symmetric stretching of P–O groups, respectively. The modes at 1034 cm^− 1^, 1044 cm^− 1^, and 1093 cm^− 1^ are attributed to the triply degenerated *ν*_*3*_ anti-symmetric stretching of the P–O groups. The bands at 565 cm^− 1^, 574 cm^− 1^, and 602 cm^− 1^ are characteristic of the triply degenerate *δ*_*4*_ bending of the O–P–O groups.

**Fig. 4:**
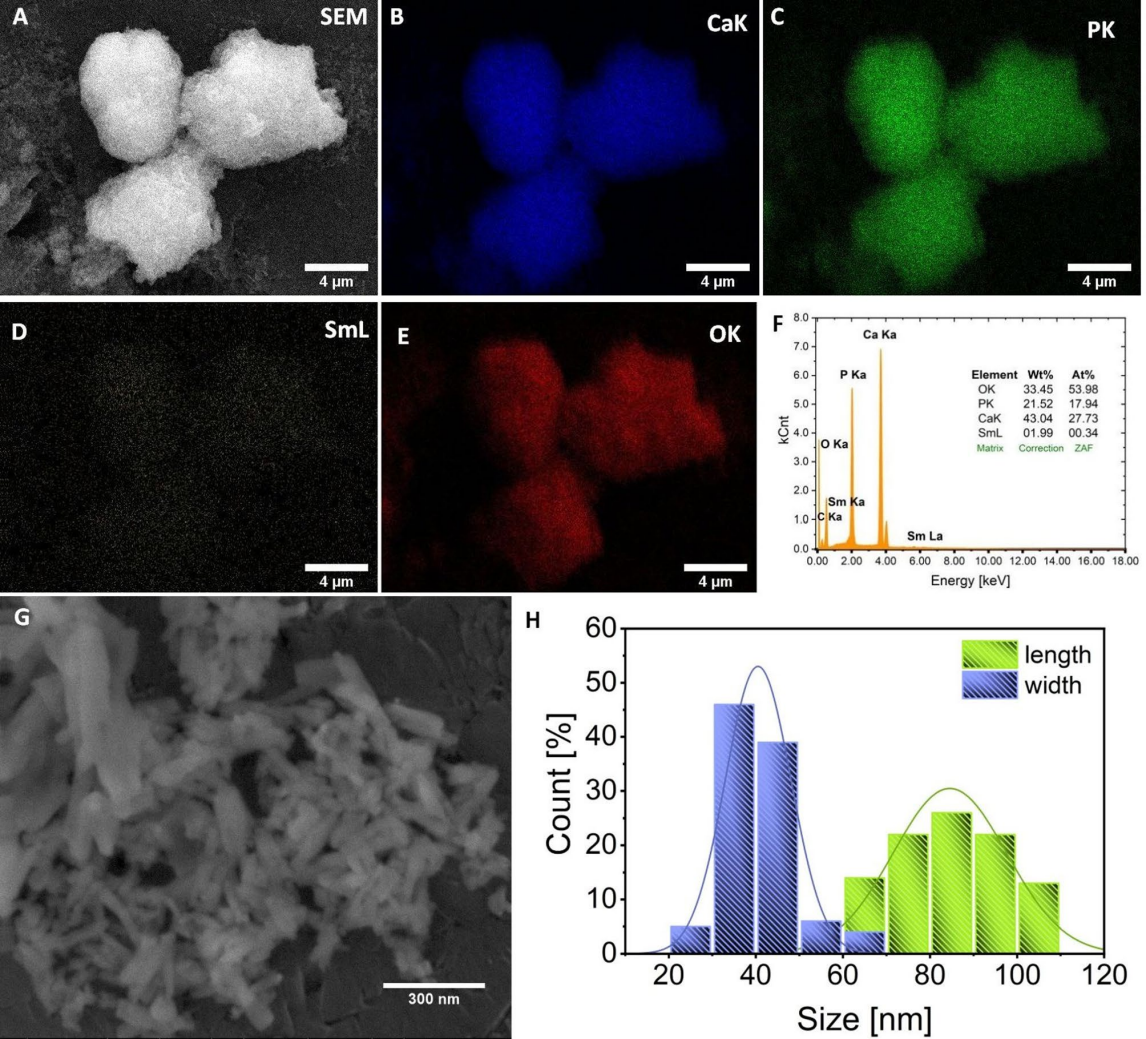
Representative SEM-EDS elemental maps (A-E) of the nHAp: 5 _mol_% Li^+^, 1 _mol_% Sm^3+^ annealed at 500 ^°^C. EDS spectrum with the results of ions content (F) in the nHAp co-doped with 5 _mol_% Li^+^ and 1 _mol_% Sm^3+^ based on SEM-EDS measurements. Representative SEM image (G) of the nHAp: 5 _mol_% Li^+^, 1 _mol_% Sm^3+^ together with histogram (H) of the grain size distribution (length-wise and width-wise diameters).

**Table 2:** Representative ICP-OES results of the 1 _mol_% Sm^3+^,5 _mol_% Li^+^:nHAp.

Sample	Li (wt%)	Sm (wt%)	Ca (wt%)	*P* (wt%)		$\:\frac{{n}_{Ca}}{{n}_{\left(Li+Ca+Sm\right)}}$ (mol%)	$\:\frac{{n}_{Li}}{{n}_{\left(Li+Ca+Sm\right)}}$ (mol%)	$\:\frac{{n}_{Sm}}{{n}_{\left(Li+Ca+Sm)\right)}}$ (mol%)	$\:\frac{{n}_{\left(Li+Ca+Sm\right)}}{{n}_{P}}$
nHAp: 1 _mol_% Sm^3+^,5 _mol_% Li^+^	0.382	1.51	38.30	18.56		93.62	5.39	0.98	1.70
	0.382	1.51	37.53	18.37		93.50	5.50	1.00	1.67
	0.382	1.51	39.07	18.74		93.75	5.29	0.96	1.72
Average^*^ (SD)						93.62 ± 0.12	5.39 ± 0.11	0.98 ± 0.02	1.70 ± 0.02

* SD – standard deviation.

The morphology and the particle size of the undoped nHAp, nHAp doped and co-doped with Li^+^ and Sm^3+^ ions, were investigated by the SEM technique. As can be seen in Fig. 4G, the particles are in nanoscale (grains with the average size being around 85 nm x 40 nm - histogram in Fig. 4H) and create a nanorod morphology. At the same time, no significant changes in morphology or grain size were observed after using dopants. Moreover, a homogeneous size distribution for all samples was observed due to their sintering at the same temperature equal to  500 ˚C. Moreover, the elemental maps (Fig. 4A-E) have shown the homogenous distribution of all ions in the studied materials.

The total amount of all elements in the obtained materials was determined by SEM-EDS measurements. Representative EDS spectrum of the 5 _mol_% Li^+^, 1 _mol_% Sm^3+^:nHAp, and the results have been presented in Fig. 4F. The estimated Sm^3+^ concentration was 1.21 _mol_% (SD = 0.04 for *n* = 3, where SD means Standard Deviation and n means number of independent measurements). The amount of Li^+^ ions in the material was estimated by the ICP-OES technique. The overall amount of Ca^2+^, Sm^3+^, Li^+,^ and P^5+^ ions has been gathered in Table 2. The content of Li^+^ and Sm^3+^ ions is equal to 5.39 ± 0.11 and 0.98 ± 0.02, respectively. This result shows a good agreement with the assumed quantity of dopants during the synthesis. Moreover, the ratio of all cations to phosphorus ions is 1.70 ± 0.02, which fits very well with the theoretical ratio of Ca/P in the hydroxyapatite structure (1.67).

### Spectroscopic characterization of the nHAp doped and co-doped with Sm^3+^ ions

**Fig. 5:**
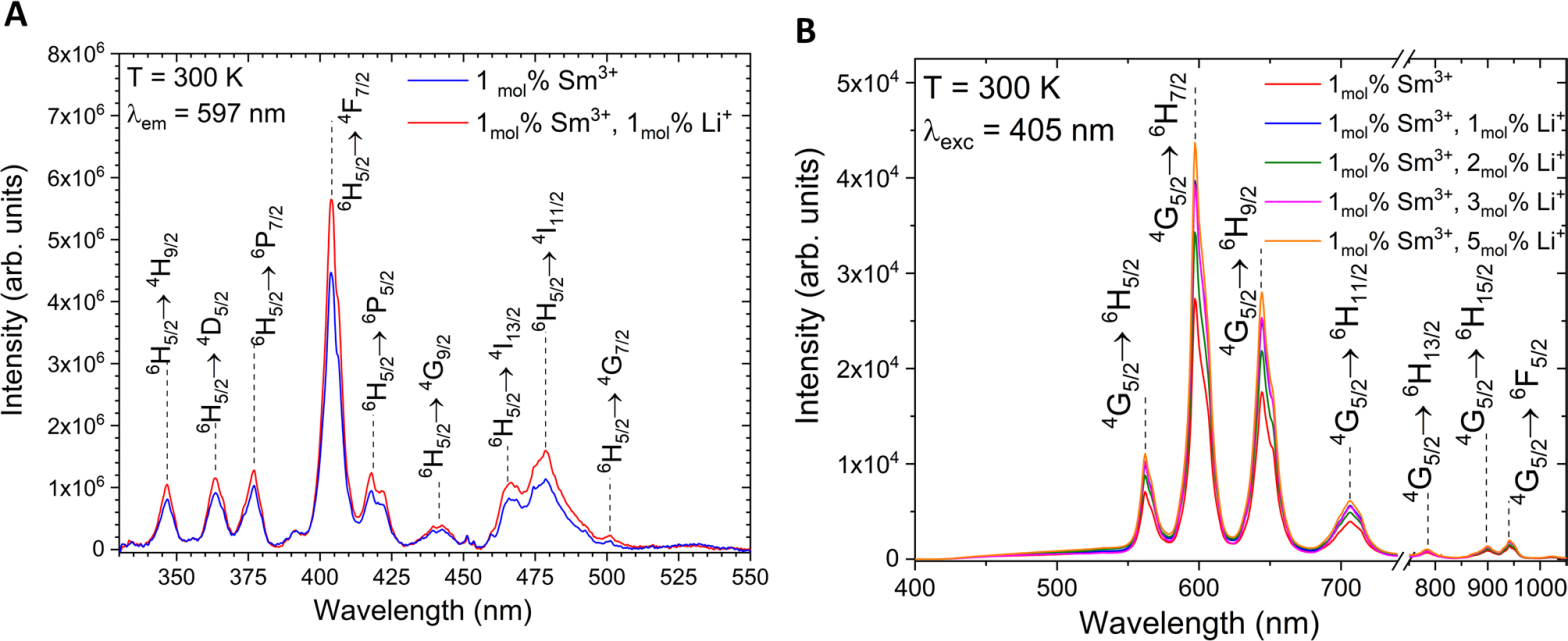
Room-temperature excitation spectra (A) of the 1 _mol_% Sm^3+^:nHAp and 1 _mol_% Sm^3+^, 1 _mol_% Li^+^:nHAp, prepared by the co-precipitation method and annealed at 500 °C. Room-temperature emission spectra (**B**) of the 1 _mol_% Sm^3+^:nHAp: and 1 _mol_% Sm^3+^, 1–5 _mol_% Li^+^:nHAp, prepared by the co-precipitation method and annealed at 500 °C.

The emission and excitation spectra of the 1 _mol_% Sm^3+^:nHAp and 1 _mol_% Sm^3+^, 1–5 _mol_%Li^+^ :nHAp shown in Fig. 5 have been measured at room temperature (300 K). All observed emission lines are typical for Sm^3+^-doped hydroxyapatites. Those attributed to the emission are: ^*4*^*G*_*5/2*_ *→* ^*6*^*H*_*J*_ (*J = 5/2*,* 7/2*,* 9/2*,* 11/2*,* 13/2*,* 15/2*) and ^*4*^*G*_*5/2*_ *→* ^*6*^*F*_*5/2*_ with the most intense transition located at 597 nm (^*4*^*G*_*5/2*_ *→* ^*6*^*H*_*7/2*_).

The ratio of the electric-dipole (^*4*^*G₅/₂ →* ^*6*^*H*_*9*_*/₂*) to magnetic-dipole (^*4*^*G₅/₂ →* ^*6*^*H₅/₂*) transitions, *R*, was used to assess the local environment of Sm³⁺ in hydroxyapatite^36^ (Table 3). The obtained values of the *R* parameter (4.31–4.52), which significantly exceed the typical values ​​observed for Sm³⁺ ions in higher symmetry environments, clearly indicate a strongly asymmetric local coordination around the Sm³⁺ ion. Co-doping with the Li⁺ ion only modifies the degree of local asymmetry, without altering the preferential site occupancy of the Sm³⁺ ion. This conclusion is supported by our earlier studies on a similar system, in which the Eu^3+^ ion was used as an optical probe to investigate the local environment and thus preferential site occupancy^23^.

**Table 3:** Asymmetry ratio (R) of the Ca_10_(PO_4_)_6_(OH)_2_ doped with Li^+^ (nHAp: Li^+^) and Sm^3+^ (nHAp: Li^+^/Sm^3+^), calculated based on the luminescence spectra.

Sample	*R* [(^4^G₅/₂ → ^6^H_9_/₂)/(^4^G₅/₂ → ^6^H₅/₂)]
nHAp: 1 _mol_% Sm^3+^	4.52
nHAp: 1 _mol_% Sm^3+^,1 _mol_% Li^+^	4.31
nHAp: 1 _mol_% Sm^3+^,2 _mol_% Li^+^	4.91
nHAp: 1 _mol_% Sm^3+^,3 _mol_% Li^+^	4.38
nHAp: 1 _mol_% Sm^3+^,5 _mol_% Li^+^	4.42

**Fig. 6:**
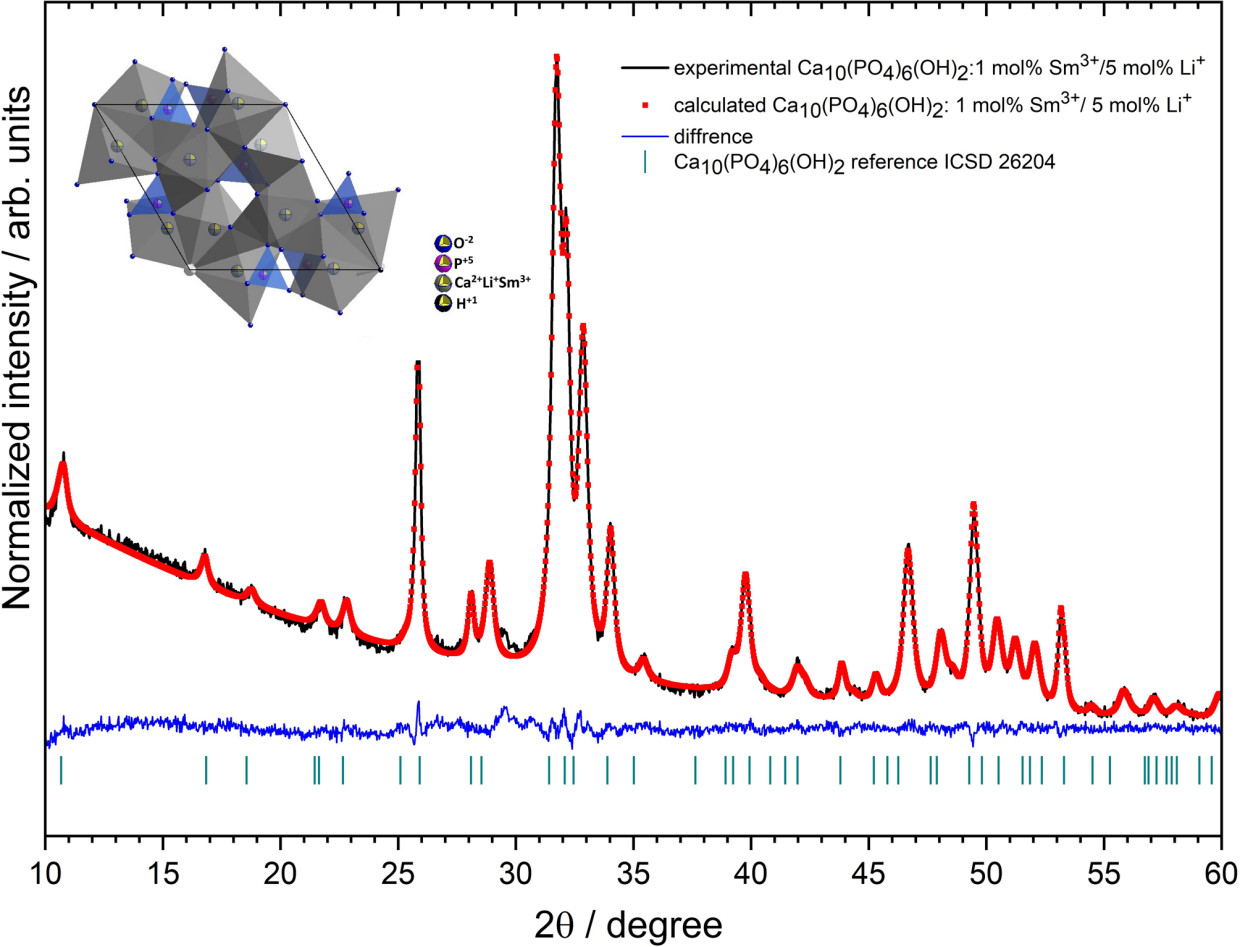
Representative results of Rietveld analysis for Ca_10_(PO_4_)_6_(OH)_2_ doped with 1 _mol_% Sm^3+^ and 5 _mol_% Li^+^ (black- experimental, red dots – fitted diffraction, blue – differential pattern, and cyan column – reference (ICSD- 26204) phase peak position).

To identify the preferential site of Sm³⁺ ion incorporation, the Rietveld refinement analysis was performed (Fig. 6). using MAUD software (version 2.9994), adopting the hexagonal apatite structure model and an improved CIF-based indexing approach. The Levenberg-Marquardt algorithm with a pseudo-Voigt peak function was used in the refinement calculations. The quality of the refinement was assessed using standard *R*-factors (*R*_*w*_, *R*_*wnb*_, *R*_*all*_, *R*_*nb*_, and *σ*), and additional parameters were introduced to increase the accuracy and reliability of the fit. The results confirm the formation of a hexagonal phase and indicate that Sm³⁺ ions successfully replace Ca^2+^ ions at two crystallographic positions (Ca_1_ and Ca_2_), with a similar distribution. As shown in Fig. 6, the calculated pattern closely matches the experimental XRPD data. Small, near-zero deviations on the difference curve (Y_obs_ − Y_calc_) further confirm the robustness of the refinement. A summary of the refinement parameters is presented in Table 4.

**Fig. 7:**
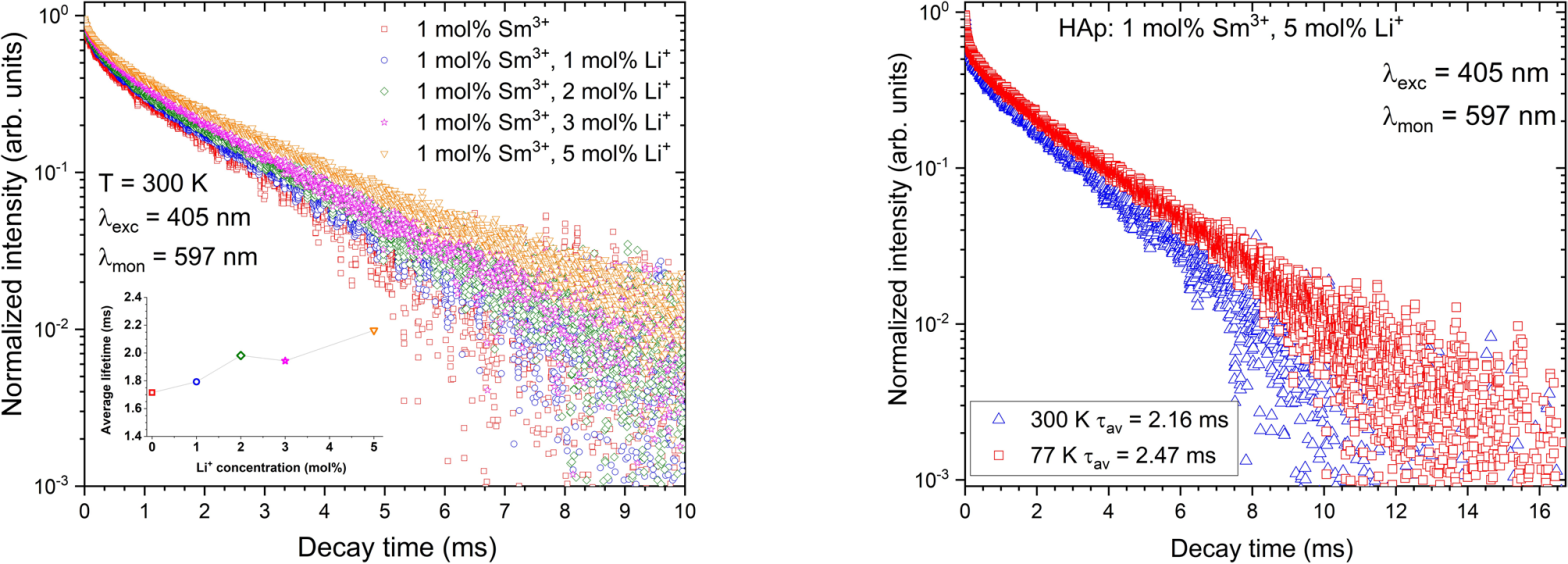
The decay profiles of nHAp: doped with Sm^3+^ and co-doped with different concentrations of Li^+^ ions, recorded at 300 K (A), and comparison with measurements at 77 K (B) for the 597 nm emission line corresponding to the ^*4*^*G*_*5/2*_ *→* ^*6*^*H*_*7/2*_ transition, with the indication of Sm^3+^ ion average lifetimes (A inset).

The luminescence decay profiles of nanohydroxyapatite (nHAp) doped with 1 _mol_% Sm³⁺ and co-doped with varying concentrations of Li⁺ ions (1–5 _mol_%) are presented in Fig. 7. The decay profiles for the emission at 597 nm originate from the *⁴G₅/₂ → ⁶H₇/₂* transition of Sm³⁺ ions. The inset shows the dependence of the average luminescence lifetime of Sm^3^⁺ ions on lithium concentration. The decay profiles (at 300 K) show non-single-exponential curves, which are characteristic of multiple radiative and non-radiative relaxation pathways. This effect may also be related to the occupation of more than one crystallographic site in the hydroxyapatite lattice (Ca_1_ and Ca_2_) by Sm³⁺ ions, which provides different local environments. The influence of other local environments, such as defect sites or lattice distortions induced by Li⁺ ion incorporation, cannot be excluded.

**Table 4:** Atomic parameters of Rietveld analysis for Ca_10_(PO_4_)_6_OH_2_ doped with 1 mol% Sm^3+^ and 5 mol% Li^+^.

Sample	Ca_10_(PO_4_)_6_ (OH)_2_: 1 _mol_% Sm^3+^, 5 _mol_% Li^+^; Z = 1					
Space group	Hexagonal *P6*_*3*_*/m* (No. 176)					
Calculated cell parameters	*a* = 9.429(6) Å c = 6.883(3) Å *V* = 529.96(71) Å^3^					
*R*_w_	2.50%					
*R*_wnb_	3.50%					
*R*_all_	1.95%					
*R*_nb_	3.62%					
σ	1.42%					
Selected shortest contacts						
Ca|Li|Sm – Ca|Li|Sm	3.9566(5) Å					
Ca|Li|Sm – O	2.3109(6) Å					
Ca|Li|Sm – P	3.2019(6) Å					
P – O	1.4878(8) Å					
Ca|Li|Sm – O – Ca|Li|Sm	100.449(2)°					

Atom	Wyckoff positions	x	y	z	*B*_*iso*_	Occ. (< 1)
O1	*6 h*	0.3336	0.4843	0.2498	0.01174	
O2	*6 h*	0.5830	0.4593	0.2495	0.01062	
O3	*12i*	0.3419	0.2593	0.0659	0.0721	
P1	*6 h*	0.4008	0.3694	0.2504	0.20601	
Ca1	*4f*	0.3330	0.6669	0.0020	0.0543	0.94593
Ca2	*6 h*	0.2443	0.9929	0.2495	0.0382	0.99407
O4	*4e*	0	0	0.0382	0.01714	0.5
H1	*4e*	0	0	0.0758	0.139996	0.5
Sm1	*4 f*	0.3330	0.6669	0.0020	0.12674	0.00508
Li1	*4f*	0.3330	0.6669	0.0020	0.12674	0.04899
Sm2	*6 h*	0.2443	0.9929	0.2495	0.66841	0.00492
Li2	*6 h*	0.2443	0.9929	0.2495	0.66841	0.00101

Moreover, the average lifetime increases with increasing Li⁺ ion concentration, and the maximum value belongs to 5 mol%. This suggests that the incorporation of Li⁺ ions induces changes in the local crystal field symmetry and thus reduces the probability of non-radiative relaxation due to the defect states. Li⁺ ions act as charge compensators, lattice stabilizers, and non-radiative defect suppressors. Li⁺ can adjust the charge balance (when the divalent calcium cation is substituted by a trivalent samarium cation). Due to its small size, Li+ can occupy not only cationic vacancies, but also interstitial positions, thereby stabilizing the crystal lattice, reducing the number of non-radiative defects, and improving the local environment for Sm³⁺ luminescence. More detailed charge-compensation mechanisms were considered in our previous studies based on the Kröger–Vink notation^8,23^. At lower temperature (77 K), the estimated lifetimes are significantly larger by approximately 0.3 ms when compared with the lifetimes detected at room temperature. It is caused by the inhibition of non-radiative processes at low temperature, allowing radiative transitions to dominate. Moreover, in both temperatures, the co-doping with Li⁺ ion leads to the trend in increasing in the average lifetime with Li⁺ ion concentration, indicating a beneficial role of Li⁺ ion in the HAp matrix.

**Fig. 8:**
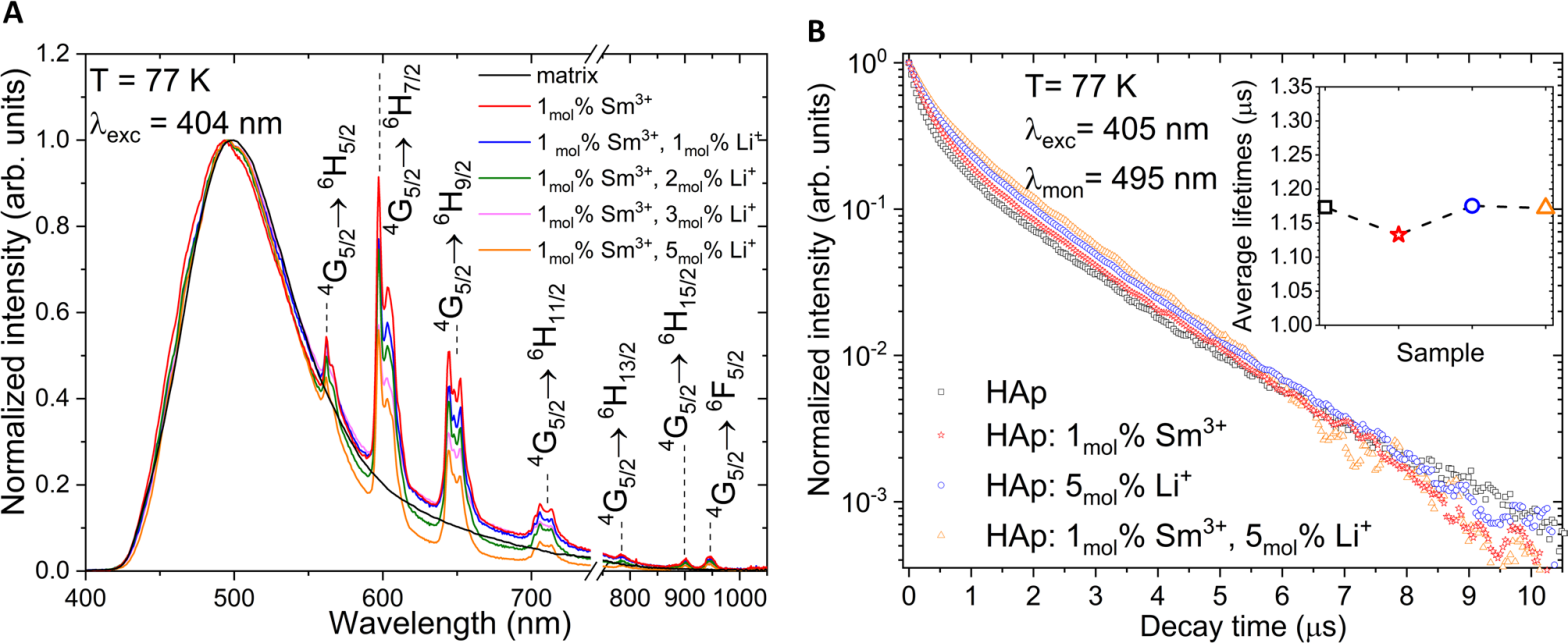
Luminescence spectra of the 1 mol% Sm^3+^:nHAp: and 1 mol% Sm^3+^, 1–5 mol% Li^+^:nHAp, measured at liquid nitrogen temperature (77 K).

In Fig. 8, the normalized luminescence spectra of samarium and lithium-doped nHAp are shown. The samples were excited at 404 nm and measured at 77 K (reduced thermal quenching). In comparison to measurements at 300 K (Fig. 5), a broad emission band in the 425–600 nm range appeared at 77 K. The detected broadband is observed for all studied materials and is not related to the type or concentration of dopants. It is also visible in the undoped HAp. It suggests the involvement of an intrinsic defect-related or host-related emissions that are enhanced at low temperatures.

**Fig. 9:**
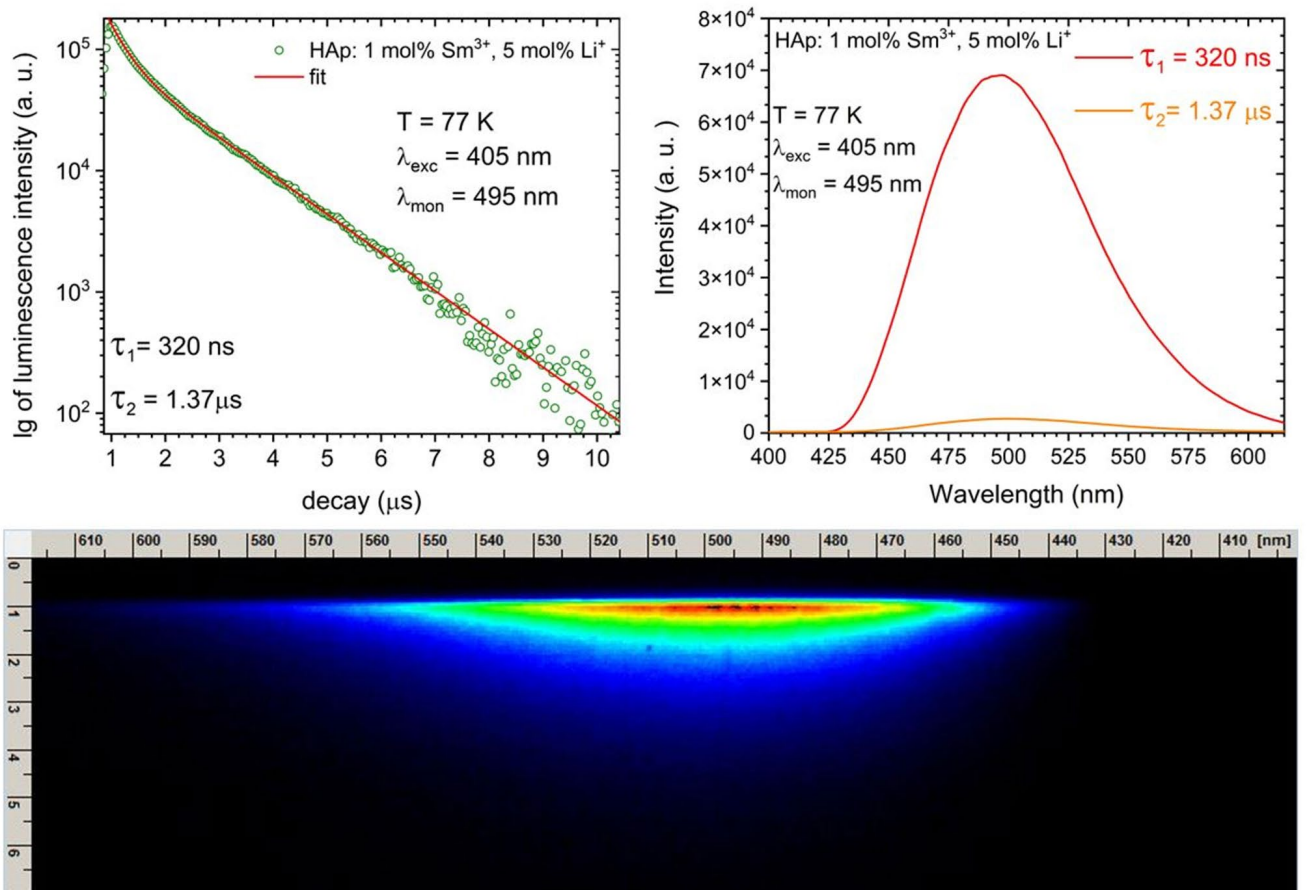
Decay profile and spectrum of the blue-green emission of nHAp: 1 mol% Sm^3+^, 5 Li^+^ mol%, measured with 405 nm pulse excitation and 495 nm observation at 77 K, detected by streak camera.

The luminescence decays of the blue-green band were measured under 405 nm pulse excitation and with the 495 nm observation. The decay curves are double-exponential with values equal to 320 ns and 1.37 µs (Fig. 9).

**Fig. 10:**
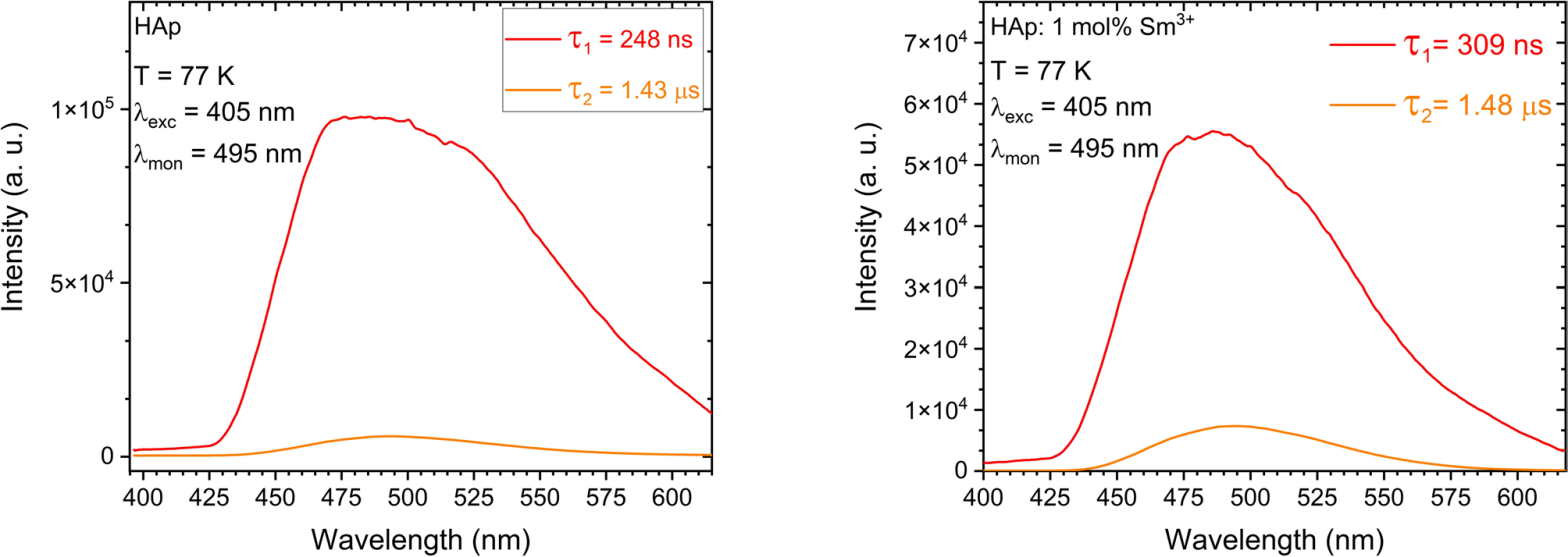
Spectra of the blue-green emission of nHAp and nHAp: 1 _mol_% Sm^3+^ measured with 405 nm pulse excitation and 495 nm observation at 77 K, detected by a streak camera with the indication of lifetimes.

Figure 10 presents the broad band spectra for the undoped material and the one doped only with Sm^3+^ ions, respectively. Comparison with the material for which data are presented in Fig. 8 confirms that Li⁺ ion co-doping causes a distinct modification of the host emission. The undoped material exhibits decay components of *τ₁* = 248 ns and *τ₂* = 1.43 µs, and a broad defect-related band centered at ~ 495 nm. The substitution of Sm³⁺ ion increases the fast decay component to *τ₁* = 309 ns and slightly changes the shape of the defect emission, indicating a modification of the native defect states. Sm³⁺/Li⁺ co-doping further alters the decay kinetics (*τ₁* = 320 ns, *τ₂* = 1.37 µs, Fig. 9) and results in a more symmetric defect band, indicating the formation of a different defect configuration. This reorganization of defect states is crucial for LIR thermometry, as the emission from defects constitutes a temperature-sensitive channel. We therefore assume that the Sm³⁺ and Li⁺ ion co-doped sample will provide a more stable, predictable, and monotonic LIR response, making it a superior thermometric material compared to a sample containing only Sm³⁺ ions.

### Temperature-dependent luminescencenanothermometry

**Fig. 11:**
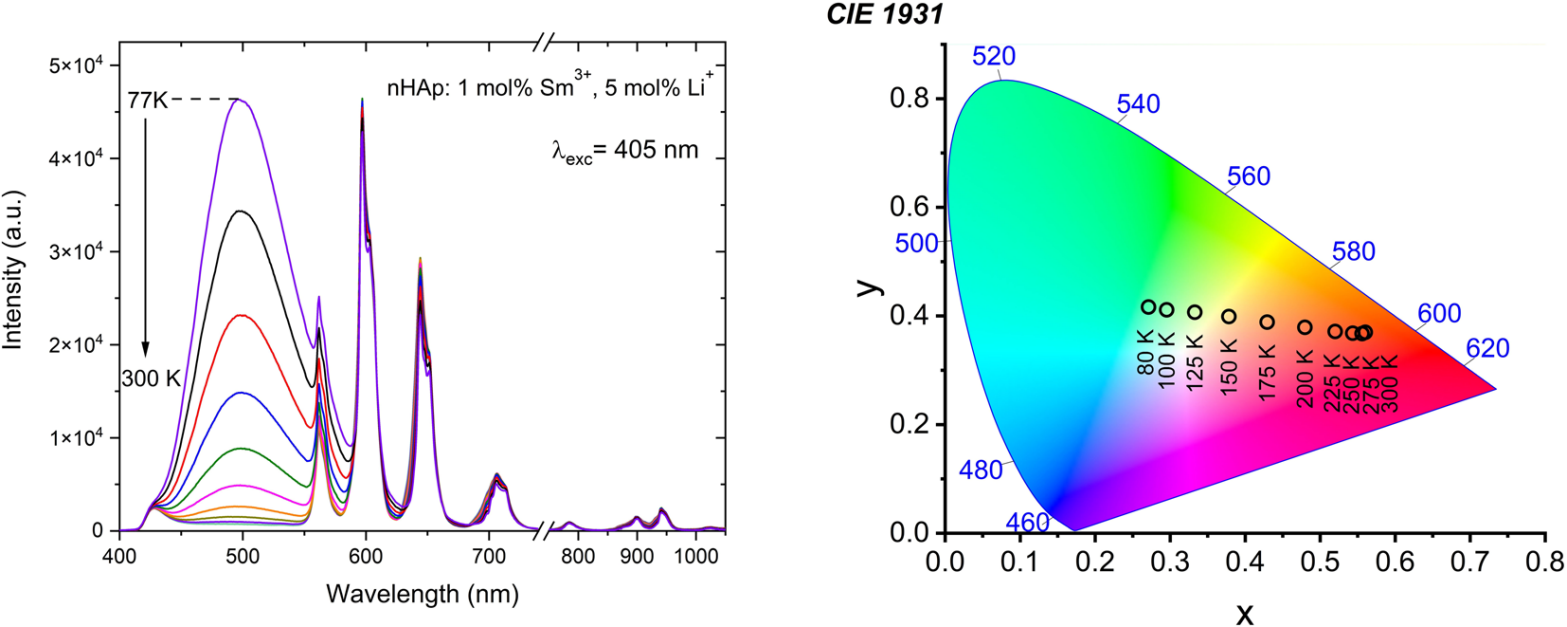
Temperature-dependent luminescence spectra of 1 _mol_% Sm^3+^, 5 _mol_% Li^+^ :nHAp (A) with the CIE chromaticity diagram (B).

Due to the observation of the broad band, we decided to perform luminescent measurements in the temperature range of 77–300 K (in 25 K intervals). The results are seen in Fig. 11 for the Sm³⁺ and Li⁺ ions co-doped nanohydroxyapatite (1 _mol_% Sm³⁺, 5 _mol_% Li⁺:nHAp) under excitation at 405 nm, from 77 K to 300 K. The spectra show strong temperature dependence of the emission intensity, particularly in the blue-green region (~ 450–550 nm). When the temperature decreases, the intensity of the broad emission band increases, reaching its maximum at 77 K. it is likely due to the suppression of non-radiative relaxation pathways below room temperature.

On the other hand, the characteristic sharp emission peaks of Sm³⁺ ions, in the orange-red region (~ 600–700 nm), show relatively stable emission through the whole temperature range, which means that these transitions are less sensitive to thermal quenching. This observation suggests that our materials have potential for temperature-independent applications.

**Fig. 12:**
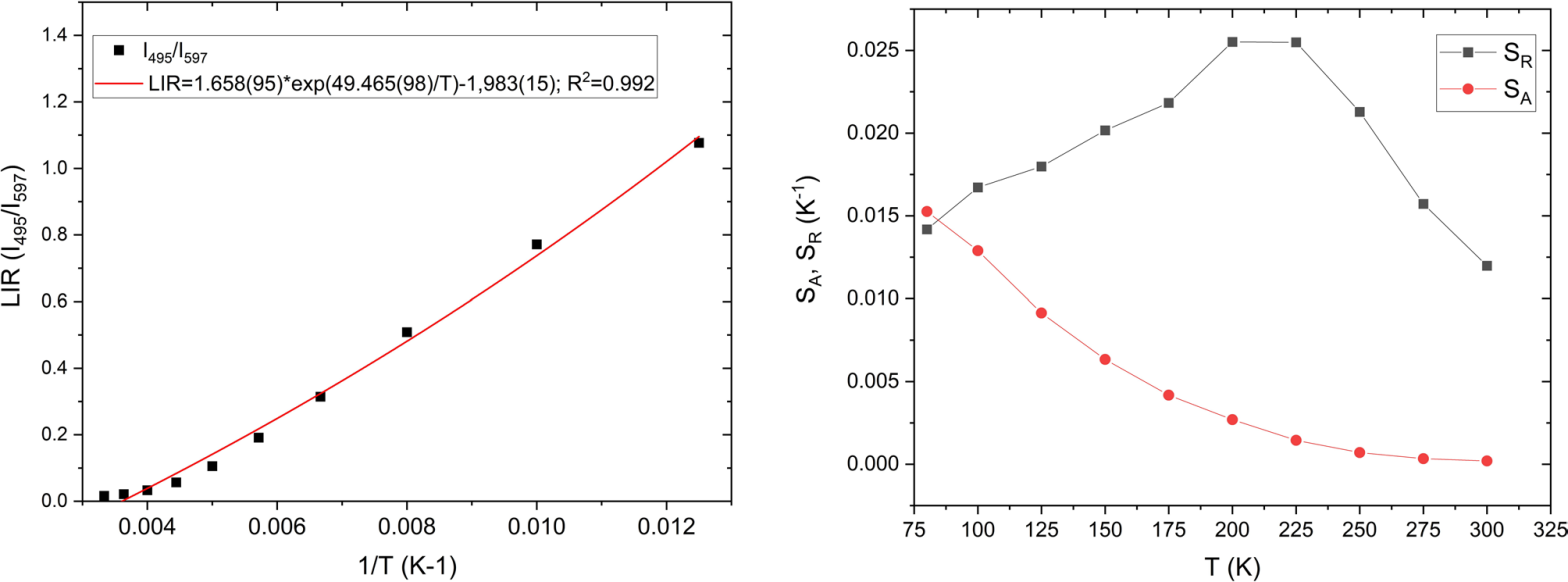
LIR vs. 1/*T* plots of *I*_green−blue_/*I*_orange_-_red_ (A) as well as variation of relative sensitivity (*S*_*R*_) and absolute sensitivity (*S*_*A*_) (B) for the 1 _mo_l% Sm^3+^, 5 _mol_% Li^+^:nHAp.

Figure 11, B presents the CIE 1931 chromaticity diagram. A nearly linear shift in emission colour is observed as temperature changes. This indicates that Sm^3+^,Li^+^:nHAp is sensitive to temperature by colorimetric readout. An intense greenish color is observed at 77 K, which is gradually shifted as the temperature increases, from yellowish at 125–150 K, orange at 175 K, to orange-red at 300 K. These results demonstrate that co-doping of nHAp by Sm^3+^ and Li⁺ ions modifies the emission of the nHAp with temperature. It makes our materials promising for optical temperature sensors. To evaluate the feasibility of using this material as a nanothermometer, we performed calculations, including the luminescence intensity ratio (LIR). Figure 12, A presents the dependence of *LIR = I*_*green−blue*_*/I*_*orange*_*-*_*red*_ on the inverse of temperature (*1/T*), to analyze thermally activated processes ( Eq. 5):

5 $$\:LIR=\frac{{I}_{blue-green}}{{I}_{orange-red}}=Aexp\left(\frac{-\varDelta\:E}{kT}\right)+C$$

where *A* is the pre-exponential parameter, *C* is a constant, *ΔE* is the activation energy, *k* is the Boltzmann constant, and *T* is the temperature at which the luminescence intensity was detected.

The parameter *ΔE* represents the activation energy in the Arrhenius-type model describing the non-radiative thermally activated quenching process that affects the blue-green emission associated with defects. In other words, *ΔE* determines how easily the defect level undergoes thermal depopulation via the non-radiative pathway according to the characteristic Arrhenius relationship exp(*−ΔE/kT*). Since the orange-red Sm³⁺ ^*4*^*G*_*₅/₂*_*→*^*6*^*H*_*J*_ f–f transitions are only weakly temperature-dependent (Sm³⁺ ion transitions are shielded by the 5s²5p⁶ configuration and thus exhibit only weak thermal quenching, acting as a stable reference), the LIR coefficient isolates thermally activated defect band behavior. The defect-related emission is strongly affected by thermally activated non-radiative relaxation, and thus its contribution to LIR follows to Arrhenius type exponential behavior. Based on our measurements, the estimated coefficient (*R²* = 0.992) indicates good agreement with the theoretical model. The *LIR* increases with decreasing temperature, which shows that temperature can be effectively read from the luminescence intensity ratio. It means that the proposed material can be applied as a temperature sensor.

Figure 12B presents the sensor’s sensitivity as a function of temperature presented as *S*_*R*_ - relative sensitivity (Eq. 6), and *S*_*A*_ - absolute sensitivity (Eq. 7).

6 $$\:{S}_{R}=\frac{1}{LIR}\frac{d\left(LIR\right)}{dT}$$

7 $$\:{S}_{A}=\frac{d\left(LIR\right)}{dT}$$

The maximum *S*_*A*_ was 0.015 K^− 1^ at 77 K and the maximum *S*_*R*_ was 0.025 K^− 1^ in the range of 200–225 K. It makes this system particularly suitable for temperature applications in cryogenic conditions – for example, in solid-state, cryobiology, and superconducting technologies.

Our Sm³⁺-activated HAp thermometric system was compared with other Sm³⁺-based luminescent thermometers. Typically, Sm³⁺-based thermometers reported in the literature achieve their highest sensitivity at high temperatures rather than at cryogenic temperatures. For example, Sm³⁺/Mn⁴⁺-doped LaGaO₃ exhibits a relative sensitivity of 4.19% K⁻¹ and 2.09% K⁻¹, but only in the temperature range of 300–500 K^24^. Similarly, Sm³⁺ ion-doped Ba₂MgMoO₆ exhibits a sensitivity of 2.7% K⁻¹ at − 30 °C (243 K) and 1.6% K⁻¹ at 75 °C, again outside the cryogenic range^25^. Ba₂ZnSi₂O₇:Sm³⁺, on the other hand, achieves a relative sensitivity of 2.02% K⁻¹, but only in the temperature range from 303 to 483 K^26^. Even co-activated Dy³⁺/Sm³⁺ Ba₂ZnSi₂O₇ exhibits very high sensitivity of 7.19% K⁻¹, but this value is achieved at 403 K and is not representative of low-temperature performance^27^. In turn, for Sm³⁺ ion-doped phosphate materials such as K₃Sc(PO₄)₂:Sm³⁺ and Ba₇Hf(PO₄)₆:Sm³⁺, no quantitative sensitivities based on LIR have been reported, and their thermometric characteristics are limited to temperatures above 300 K^28,29^. Therefore, to our knowledge, there are no Sm³⁺ ion-activated phosphates or oxides that would show high sensitivity under cryogenic conditions. Our measured values of *S*_*A*_ = 0.015 K⁻¹ at 77 K and *S*_*R*_ = 0.025 K⁻¹ at 200–225 K are therefore fully consistent with the expected behavior of defect-assisted Sm³⁺ ion thermometry at low temperatures and show that our system operates in a temperature range not accessible to other Sm³⁺ ion-based thermometric materials.

At the same time, the change in the luminescence color of 1 _mo_l% Sm^3+^, 5 _mol_% Li^+^:nHAp, depending on temperature, can be used for precise temperature monitoring at the cellular level. Our materials, as luminescent nanothermometers, enable real-time monitoring of temperature variations with high accuracy. It is worth noting that cryopreservation processes allow for precise tracking of freezing and thawing cycles, increasing the survival rate of cells and tissues after thawing and improving the overall efficiency of cryogenic techniques. Cell freezing during cryoprotection usually proceeds in several stages, and the temperature ranges depend on the cell type and the method used. At temperatures between 200.15 K and 225.15 K (-73 °C to -48 °C), our material showed the highest relative sensitivity, corresponding to the critical deep-freezing range where the risk of cell damage from ice crystal formation is greatest. Therefore, precise temperature monitoring in this range is essential for effective cryopreservation^4^.

### Mechanism of thermal quenching

**Fig. 13:**
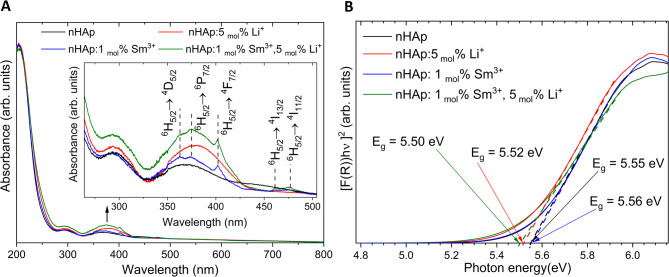
Diffuse reflectance UV-Vis absorption spectra (A) of the nHAp, 5 _mol_% Li^+^:nHAp, 1 _mol_% Sm^3+^:nHAp: ,and 1 _mol_% Sm^3+^, 5 _mol_% Li^+^:nHAp, prepared by the co-precipitation method and annealed at 500 °C. Kubelka-Munk plot (B) with the indication of band-gap energy of nHAp, 5 _mol_% Li^+^:nHAp:, 1 _mol_% Sm^3+^:nHAp:, and 1 _mol_% Sm^3+^, 5 _mol_% Li^+^:nHAp.

The thermal quenching of the broad blue-green emission band (450–550 nm) (Fig. 11) is strongly correlated with the defect-related optical behaviour of nHAp matrix. This broadband emission originates from electronic transitions involving several localized energy states within the band gap^5^. This is due to the fact that the excitation wavelength (405 nm, corresponding to 3.06 eV) is significantly lower than the optical band gap energy (Eg ≈ 5.50–5.56 eV). These values were estimated based on the UV-Vis diffuse reflectance spectra (Fig. 13A), and the Kubelka-Munk plots as shown in Fig. 13B. The presence of mid-gap defect states is responsible for the recombination of the e’-h· pair^30^ and are mostly created by structural imperfections and symmetry breaking in the [PO₄]³⁻ clusters (changes in the P–O bond lengths and O–P–O bond angles^31^) due to the doping process, also induced by defects such as OH⁻, Ca vacancies, or oxygen vacancies as radiative centers^32,33^. At low temperatures (77 K), the radiative recombination through these defect states is promoted (inhibition of non-radiative phonon interactions). However, as the temperature increases, thermal vibrations of the crystal lattice activate non-radiative relaxation pathways, and the emission intensity decreases. The thermal quenching is clearly visible for the broad defect-related emission, while the sharp Sm³⁺ ions’ *4f–4f* transitions remain relatively stable (shielded from lattice interactions).

All these results demonstrate that the optical properties of Sm³⁺/Li⁺ co-doped nHAp are strongly associated with the defect-mediated processes, which were confirmed by luminescence and UV-Vis absorption studies. The possibility of adjusting emissions through temperature and doping suggests potential applications of our materials in optical thermometry and as phosphors with engineered defects^15,34^.

## Conclusions

In this work, we proposed a method for synthesizing biomaterials intended for cryo-applications. We have found the temperature-dependent variations in emission colour using CIE chromaticity diagrams. Sm³⁺, Li^+^ ions-doped nHAp shows tunable emission shifting from blue-green to orange-red as the temperature changes from 77 K to 300 K. We concluded that it is strongly influenced by defect-mediated processes. Our material exhibited a maximum absolute sensitivity of 0.015 K⁻¹ at 77 K with the highest relative sensitivity of 0.025 K⁻¹ within the 200–225 K range. These calculations highlight the strong potential of the obtained materials for precise temperature sensing under cryogenic conditions^35^.

## Data Availability

The datasets analyzed during the current study are available from the corresponding author on reasonable request.
